# Enhancing Integrated Treatment Programs for Severe Concurrent Substance Use and Mental Disorders: Insights on Overdose from the ROAR CANADA Project: Améliorer les programmes de traitement intégré pour les troubles mentaux et les troubles liés à l’usage de substances psychoactives graves et concomitants : aperçu de la problématique des surdoses dans le cadre du projet ROAR CANADA

**DOI:** 10.1177/07067437251315516

**Published:** 2025-02-03

**Authors:** Christian G. Schütz, Tonia Nicholls, Laura Schmid, Sydney Penner, Myra Massey, Karina A. Thiessen, Stefanie Todesco, Reza Rafizadeh, Kiefer Cowie, Sabrina K. Syan, James MacKillop

**Affiliations:** 1 Department of Psychiatry, 12358 Faculty of Medicine, University of British Columbia, Vancouver, British Columbia, Canada; 2 60299 BC Mental Health and Substance Use Services Research Institute, Provincial Health Services Authority, Vancouver, British Columbia, Canada; 3 Peter Boris Centre for Addictions Research, St. Joseph's Healthcare Hamilton/McMaster University, Hamilton, Ontario, Canada; 4 Department of Psychiatry and Behavioral Neurosciences, 3710 McMaster University, Hamilton, Ontario, Canada

**Keywords:** dual diagnosis, integrated treatment, overdose, evidence-based medicine, health disparities, inpatient psychiatry, mental health services, risk factor

## Abstract

**Objective:**

This paper summarizes methods and initial overdose-related results from the Reducing Overdose and Relapse: Concurrent Attention to Neuropsychiatric Ailments and Drug Addiction (ROAR CANADA) project. ROAR CANADA is a longitudinal observational study of individuals with severe concurrent substance use and mental disorders (also called dual disorders or dual diagnosis). The study sampled patients treated at two tertiary treatment centres in British Columbia, Red Fish Healing Centre and Heartwood Centre, along with a concurrent treatment unit at St. Joseph's in Ontario. These facilities have implemented evidence-based integrated treatment programs. Our first analysis explores selected baseline characteristics as potential risk factors for drug overdose in this population.

**Method:**

Sociodemographic factors, trauma history, and impulsivity were part of a more comprehensive longitudinal assessment. In this first investigation, we use bivariate analysis and logistic and linear regression modelling to examine these variables in relation to overdose history.

**Results:**

Overall, 291 of 450 participants (64.7%) reported a history of ≥1 overdose. Across the three centres, patients had a lifetime average of 7.6 (*SD* = 12.9) overdoses. The prevalence and mean number of overdoses were somewhat higher among Red Fish patients (74.5% and 8.5, respectively). Adverse childhood events, lifetime trauma history, and impulsivity were all high, but only lifetime trauma history emerged as significantly associated with overdose across all treatment centres. Impulsivity indicators were selectively associated with overdose by site, but not consistently within the overall sample.

**Conclusions:**

These results highlight the importance of prioritizing trauma-informed care in the treatment of individuals with severe concurrent substance use and mental disorders, who are at high risk of overdose. The integration of trauma treatment into existing programs may enhance patient outcomes and contribute to the ongoing evolution of effective care strategies for this complex population. These findings are particularly relevant in light of the overdose crisis.

**Plain Language Summary Title:**

Enhancing Integrated Treatment Programs for Severe Concurrent Substance Use and Mental Disorders: Insights on Overdose from the ROAR CANADA Project

## Plain Language Summary

Objective: This paper presents the methods and initial findings from the Reducing Overdose and Relapse: Concurrent Attention to Neuropsychiatric Ailments and Drug Addiction (ROAR CANADA) project. ROAR CANADA is a longitudinal study of individuals with severe substance use and severe mental disorders (concurrent or dual disorders). The study included patients from two BC treatment centers, Red Fish Healing Centre and Heartwood Centre, as well as a treatment unit at St. Joseph's in Ontario, all of which use a comprehensive integrated treatment program. Our initial analysis focused on identifying transdiagnostic risk factors for drug overdose.

Method: We assessed sociodemographic factors, trauma history, and impulsivity as part of a broader longitudinal evaluation. Using bivariate analysis and logistic and linear regression models, we examined the relationship between these factors and overdose history.

Results: Out of 450 participants, 291 (64.7%) reported having experienced one or more overdoses, with an average of 7.6 overdoses per person. Patients at Red Fish had a slightly higher average (8.5 overdoses) and prevalence (74.5%) of overdoses. High rates of adverse childhood events, lifetime trauma, and impulsivity were observed. However, only lifetime trauma was consistently associated with overdose risk across all treatment centers. Impulsivity was linked to overdose risk in some locations but not across the entire sample.

Conclusions: The findings underscore the importance of trauma-informed care in treating individuals with severe concurrent substance use and severe mental disorders.

## Résumé

**Objectif:**

le présent article résume les méthodes utilisées dans le cadre du projet « Reducing Overdose and Relapse: Concurrent Attention to Neuropsychiatric Ailments and Drug Addiction » (ROAR CANADA), ainsi que les résultats du projet relatifs aux surdoses. ROAR CANADA est une étude observationnelle longitudinale menée auprès de personnes souffrant de troubles mentaux et de troubles liés à l’usage de substances psychoactives graves et concomitants (également appelés « double pathologie » ou « diagnostic double »). L’étude était fondée sur un échantillon de patients traités dans deux centres de traitement de troisième ligne de la Colombie-Britannique, le Red Fish Healing Centre et le Heartwood Centre, ainsi que dans une unité de traitement des troubles concomitants du centre de soins de santé St. Joseph's, en Ontario. Ces établissements ont mis en œuvre des programmes de traitement intégré fondés sur des données probantes. Notre première analyse examine certaines caractéristiques de base considérées comme des facteurs de risque potentiels de surdose de drogue dans cette population.

**Méthode:**

les facteurs sociodémographiques, les antécédents de traumatisme et l’impulsivité ont fait l’objet d’une évaluation longitudinale plus complète. Dans cette première phase, nous utilisons une analyse bivariée et un modèle de régression logistique et linéaire pour examiner ces variables en relation avec les antécédents de surdose.

**Résultats:**

en tout, 291 des 450 participants (64,7%) ont déclaré avoir déjà fait au moins une surdose. Dans les trois centres, les patients avaient subi en moyenne 7,6 surdoses (écart-type = 12,9) au cours de leur vie. La prévalence et le nombre moyen de surdoses étaient un peu plus élevés chez les patients du Red Fish (74% et 8,5%, respectivement). Les expériences négatives de l’enfance, les traumatismes subis au cours de la vie et l’impulsivité étaient tous élevés, mais seuls les traumatismes subis au cours de la vie sont apparus comme significativement associés à une surdose dans tous les centres de traitement. Les indicateurs d’impulsivité ont été sélectivement associés aux surdoses par établissement, mais pas de manière constante dans l’ensemble de l’échantillon.

**Conclusions:**

ces résultats soulignent l’importance de privilégier les soins tenant compte des traumatismes dans le traitement des personnes souffrant de troubles mentaux et de troubles liés à l’usage de substances psychoactives graves et concomitants, ces personnes présentant un risque élevé de surdose. L’intégration du traitement des traumatismes dans les programmes existants peut améliorer l’état de santé des patients et contribuer à l’évolution continue des stratégies de soins efficaces pour cette population complexe. Ces résultats sont particulièrement pertinents à la lumière de la crise des surdoses.

## Introduction

Canada's overdose crisis is driven by the widespread presence of opioids^1–3^ and rising use of stimulants,^4^ particularly among marginalized groups.^5,6^ Canada has among the highest rates of overdose-related deaths internationally.^7–9^ Individuals with concurrent mental health and substance use disorders (SUDs) face a significantly elevated risk of fatal and non-fatal overdose compared to those with only one disorder.^6,10–13^ Despite increased attention paid to concurrent disorders,^14–20^ gaps in care remain for people with concurrent severe SUDs and severe mental disorders (or severe concurrent disorders [SCD]).^21^

Severity of concurrent disorders is not well-defined; clinically, it is characterized by number of disorders, capacity for engagement and psychosocial functioning, as well as risk of crisis, marginalization, and/or inadequate care in primary, secondary, and even tertiary treatment. Individuals with SCD are regularly underrepresented or missing in concurrent disorder studies. They are difficult to recruit and need substantial support to complete assessments, particularly when addressing the breadth and depth needed to describe their complex profile. Literature that does exist is limited by small sample sizes.^22,23^

The Reducing Overdose and Relapse: Concurrent Attention to Neuropsychiatric Ailments and Drug Addiction (ROAR CANADA) study follows SCD clients from three inpatient treatment centres. All three programs were developed in recent years and have implemented comprehensive, evidence-informed, integrated treatment and recovery programs: two tertiary programs in British Columbia (Red Fish Healing Centre and Heartwood Centre) and one specialized unit at St. Joseph's in Hamilton, Ontario.^13^

ROAR CANADA is designed to help understand the risks and needs of this vulnerable population, particularly as it pertains to overdose. We report study design, method, and baseline data, prioritizing overdose as our initial outcome of interest. Prior studies at the Red Fish Healing Centre's predecessor, the Burnaby Centre for Mental Health and Addiction, indicated that 74% of patients had a history of overdose, averaging ∼7 lifetime overdoses per person.^24,25^ High rates have been observed clinically at St. Joseph's Healthcare Hamilton. This baseline paper focuses on two major transdiagnostic risk factor domains, which have been associated with almost all disorders and with overdose in less complex populations – namely trauma history^26,27^ and impulsivity.^28–31^ A secondary focus was exploring sex-based differences in these factors. Transdiagnostic factors are not only important to understand SCD, but may also open venues for developing more effective and efficient interventions for SCD.^32^ A tertiary aim was to conduct a cross-site comparison to identify site-specific differences and patterns.

Trauma and adverse childhood experiences have been reported to increase risk of all mental disorders, including SUDs.^33,34^ Impulsivity is similarly elevated in most mental disorders and SUDs, with the relationship being less clear in severe mental disorders.^31,35,36^ Suffering from mental disorders, including SUDs, increases the risk of experiencing overdoses, but the evidence is again lacking for severe mental disorders.^37^ There is some evidence that impulsivity may be associated with increased overdose risk, particularly in polysubstance use disorders.^38^ Likewise, there is some evidence that trauma history, including adverse childhood experiences, increases risk of overdose in people who use drugs.^39,40^ However, in both cases, literature on SCD is missing. To the best of our knowledge, this is the first study on trauma history and impulsivity in relation to overdose risk within a SCD sample.

## Methods

### Settings and Samples

Participants were inpatients at St. Joseph's Healthcare Hamilton (St. Joseph's), Red Fish Healing Centre for Mental Health and Addiction (Red Fish), or Heartwood Centre for Women (Heartwood). Red Fish and Heartwood are long-term stabilization, treatment and recovery centres, accepting individuals from across BC who have exhausted local resources. St. Joseph's is a specialized concurrent disorder acute psychiatry unit, with referrals from psychiatric emergency services. During admission, patients in all three sites received comprehensive integrated services for psychiatric stabilization, SUD, mental disorder psychoeducation and psychotherapy, and group-based recovery programming.

To ensure inclusivity and representativeness, minimal inclusion criteria were set. Eligible participants had to be at least 19 years old, current treatment clients at one of the three centres, deemed sufficiently stable by staff, willing to grant access to administrative records for longitudinal analysis, fluent in English, able to provide informed consent (assessed by a seven-item quiz), and have no known discharge plans for the following month.

### Recruitment and COVID-19 Impact

Initially, participants were recruited via talks at group activities and flyers posted on bulletin boards and digital displays. As the study progressed, the need for more active in-person recruitment became evident, which involved informal one-on-one conversations with researchers.

The research team encountered significant challenges due to the COVID-19 pandemic. Research activities were curtailed early on, as non-essential workers were not permitted in the centres due to the high risk for disease transmission at that time. In addition, intermittent occurrences of cases in the centres resulted in periodic restrictions on access to individual units and, at times, the entire facility.

All procedures were approved by the University of British Columbia Clinical Ethics Review Board. Permission was granted by BC Mental Health and Substance Use Services in BC, and the Hamilton Integrated Research Ethics Board in ON.

### Data Sources and Procedures

The broader study aims to comprehensively understand individuals with SCD in Canada, focusing on relapse and overdose rates during, before, and after treatment.^41,42^ Data are collected from self-report questionnaires, medical charts, and will be complemented by provincial administrative records. Participants complete web-based questionnaires at treatment centres, with baseline assessments conducted when clients are clinically stable. Follow-up data are collected at 1–2 weeks pre-discharge, and 1- and 6-month post-discharge. Data from medical charts include psychiatric diagnoses, medical history, medications, mental health status, criminal justice involvement, and substance use. This paper focuses on understanding overdose by examining the relationship between transdiagnostic characteristics of impulsivity and trauma history and lifetime self-reported overdose.

### Measures

#### Sociodemographics

Participants self-reported age, gender identity, assigned sex at birth, race, ethnicity, living circumstances, relationship status, sexual orientation, number of children, level of education for themselves and their parents, household financial circumstances and household income, employment status, and access to a primary care physician.

#### Primary Outcome: Overdose History

At baseline, participants’ overdose histories were evaluated using two questions, (1) *Have you ever overdosed in your life?* (yes/no). If participants endorsed an overdose history, they were presented with a follow-up question, (2) *How many times have you overdosed?* (open text box requiring integers).

#### Transdiagnostic Domains: Trauma and Impulsivity

Participants completed questionnaires assessing trauma and adversity and impulsivity at baseline. Childhood trauma was measured by the Adverse Childhood Experiences (ACE) Questionnaire,^43^ and lifetime trauma was captured by the Brief Trauma Questionnaire (BTQ).^44^ Measures of impulsive personality traits (Shortened Urgency-Premeditation-Perseverance-Sensation Seeking-Positive Urgency Impulsive Behavior Scale [S-UPPS-P]),^45^ as well as impulsive decision making, also referred to as delay discounting (Monetary Choice Questionnaire [MCQ]) were also administered.^46^

### Data Analysis

Data underwent evaluation for missing values, normality, and outliers. Participants who did not respond to the overdose questionnaire were excluded. A quality control method was applied via the MCQ by excluding participants who answered both control questions incorrectly (larger vs. smaller rewards, both available immediately). Log10 transformations were applied to correct positive skewness in both MCQ scores and the total number of overdoses. Analyses were conducted using combined data from all three sites. Between-group analyses were conducted on demographic, predictor, and outcome variables to compare participants who reported a history of overdose and those who did not. Independent samples *t*-tests were used to compare continuous variables while categorical variables were compared using the chi-square test. Bivariate correlations were conducted with all variables to examine zero-order associations and to assess for multicollinearity. Multivariate logistic regression models were used to assess predictors of any history of overdose compared to no overdose. Multivariate linear regression models were used to assess predictors of the number of lifetime overdoses (using the log10 transformed variable). Sociodemographic, trauma, and impulsivity variables were included in simple logistic and linear regression models. Nonsignificant individual predictor variables were excluded from multivariable logistic and linear regression models to enhance parsimony. Given the limited research in this domain, no correction for multiple comparisons was implemented to avoid type 2 errors. To explore differences between sites, demographic and predictor variables were included in separate logistic (overdose vs. no overdose history) and linear regression (number of overdoses) models. Nonsignificant individual variables were excluded from multivariate logistic and linear regression models for parsimony. To explore sex-based differences, moderation analyses were conducted using the total sample. Each moderation model included one predictor variable, sex as a moderator, and overdose (history and number) as an outcome.

## Results

### Total Sample

#### Demographics

Participant characteristics and descriptive statistics of outcomes are detailed in Table 1. A total of 450 people (*n* = 203, 45.1% male; *n* = 247, 54.9% female) participated in the study (*n*_Red Fish_ = 231, *n*_Heartwood_ = 116, and *n*_St. Joseph’s_ = 103). The mean age was 35.4 years (*SD* = 10.9), and 58.4% identified as White. A total of 64.7% (*n* = 291) reported experiencing an overdose, many participants reported multiple overdoses (*M* = 7.6, *SD* = 12.9).

**Table 1.** Participant Characteristics.

	All participants (*N* = 450)	No overdose history (*n* = 159)	History of overdose (*n* = 291)	*t*	*p*	Cohen's *d*/ϕ
Demographics						
Age	35.4 (10.9)	34.1 (10.8)	36.1 (10.9)	−1.85	0.07	−0.18
Biological sex (% female)	54.9%	51.6%	56.7%	1.09	0.30	0.05
Gender (% cisgender)	92.3%	94.3%	91.8%	1.57	0.21	0.06
Ethnicity (% European/white)	58.4%	62.3%	56.4%	1.48	0.22	0.06
Education (% university/college)	40.2%	41.5%	39.5%	0.17	0.68	−0.02
Financial status (% difficulty paying bills)	32.8%	28.5%	35.2%	2.08	0.15	−0.07
Trauma and adversity						
Traumatic exposure (BTQ)	3.8 (2.2)	3.33 (2.2)	4.14 (2.2)	−3.53	<0.01	−0.37
ACE	4.6 (2.8)	4.19 (2.7)	4.85 (2.8)	−2.37	0.02	−0.24
Impulsivity						
Negative urgency (S-UPPS-P)	11.2 (3.0)	11.0 (3.0)	11.4 (2.9)	−1.18	0.24	−0.13
Lack of perseverance (S-UPPS-P)	12.4 (2.3)	12.3 (2.3)	12.4 (2.3)	−0.57	0.57	−0.06
Lack of premeditation (S-UPPS-P)	12.1 (2.5)	12.2 (2.4)	12.0 (2.5)	0.60	0.55	0.07
Sensation seeking (S-UPPS-P)	11.0 (2.9)	10.6 (3.1)	11.2 (2.7)	−1.94	0.05	−0.21
Positive urgency (S-UPPS-P)	9.9 (3.0)	9.6 (3.1)	10.0 (3.0)	−1.22	0.22	−0.13
Delay discounting ($100)	−1.5 (1.2)	−1.6 (1.2)	−1.4 (1.3)	−1.54	0.12	−0.17
Delay discounting ($1,000)	−1.8 (1.1)	−1.9 (1.1)	−1.8 (1.1)	−0.29	0.78	−0.03

*Note.* BTQ = Brief Trauma Questionnaire; ACE = Adverse Childhood Experience Questionnaire; S-UPPS-P = Shortened Urgency-Premeditation-Perseverance-Sensation Seeking-Positive Urgency Impulsive Behavior Scale.

#### Predicting Overdose

Bivariate analyses to compare participants with and without a history of overdose on demographic variables, measures of trauma and adversity, and impulsivity (Table 1) revealed no significant differences in demographics. Significant differences were observed between groups on measures of trauma and adversity (ACE and BTQ) and on one measure of impulsivity (S-UPPS-P sensation seeking). An item-level comparison of trauma exposure between individuals who reported history of overdose and those who did not can be found in Supplementary Table 1.

**Figure 1.**
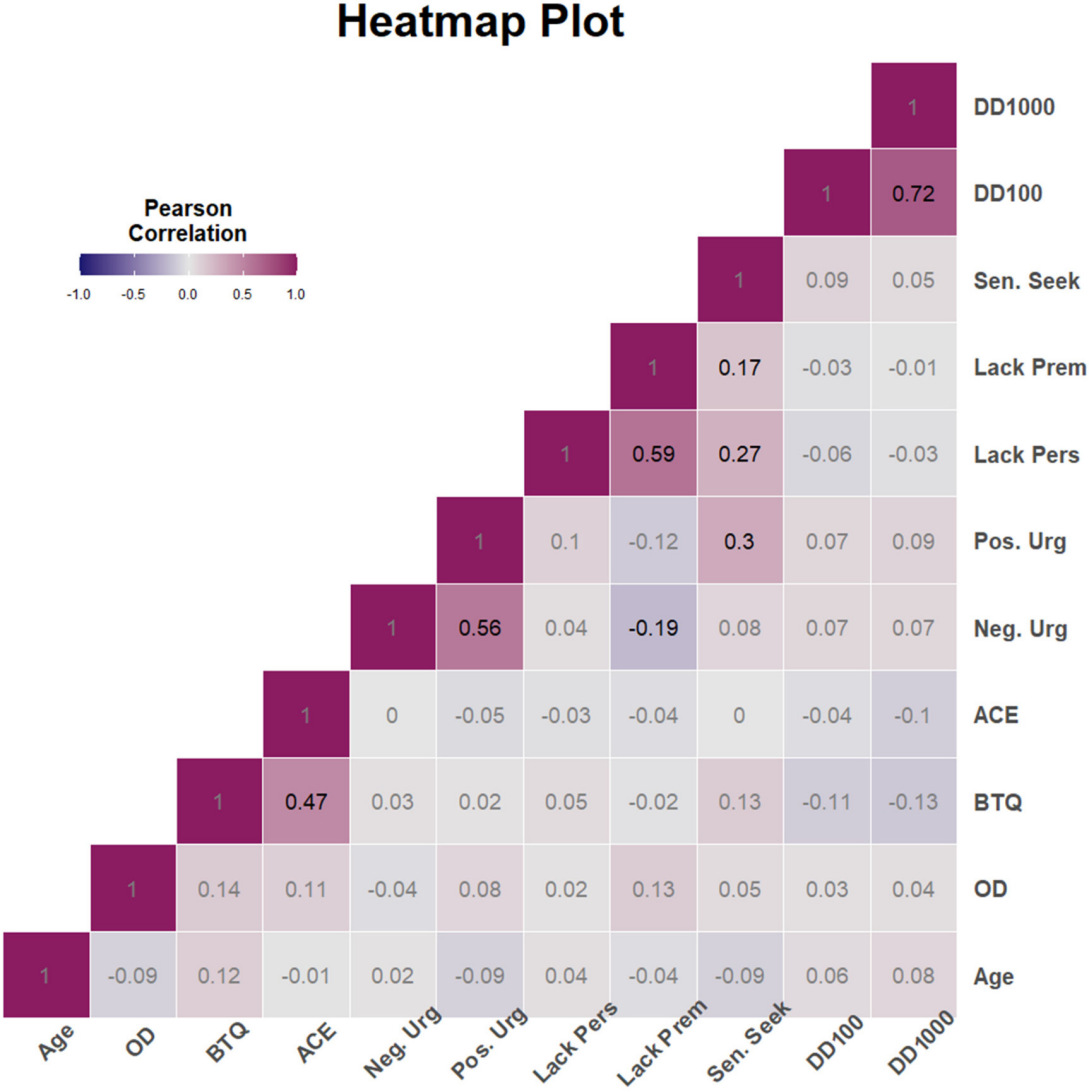
Heatmap of bivariate associations. *Note.* Heatmap of bivariate associations between age, number of lifetime overdoses (log-transformed OD), trauma and adversity measures (BTQ, ACE), impulsivity personality traits (S-UPPS-P Pos. Urg, Sen. Seek, Lack Prem, Lack Pers, and Neg. Urg), and delay discounting (DD100, DD1,000). BTQ = Brief Trauma Questionnaire; ACE = Adverse Childhood Experience Questionnaire; S-UPPS-P = Shortened Urgency-Premeditation-Perseverance-Sensation Seeking-Positive Urgency Impulsive Behavior Scale; Pos. Urg = positive urgency; Sen. Seek = sensation seeking; Lack Prem = lack of premeditation; Lack Pers = lack of perseverance; Neg. Urg = negative urgency; DD = delay discounting.

A bivariate correlation matrix (Figure 1) highlighted strong positive correlations between both MCQ variables, delay discounting $100 and delay discounting $1,000 (*r* = 0.72, *p* < 0.01). The magnitude of association among other variables varied from negligible to moderate.

In the bivariate logistic regression, lifetime trauma exposure (*B* = 0.17, *p* < 0.01), ACEs (*B* = 0.09, *p* = 0.02), and sensation seeking (*B* = 0.07, *p* = 0.05) emerged as significant individual predictors of overdose. Combining these variables into one multivariate model predicted 66.3% of group status correctly (Cox and Snell *R*^2^ = 0.03, Nagelkerke *R*^2^ = 0.04) (Table 2). Lifetime trauma exposure was the only predictor that remained significant in the multivariate model.

**Table 2.** Predictors of Experiencing an Overdose in Entire Sample Using a Binary Logistic Regression.

	*B*	*SE*	*OR*	Wald χ^2^	*p*	95% CI
Individual predictors^a^						
Age	0.02	0.01	1.02	3.38	0.07	[1.00, 1.04]
Sex	−0.21	0.20	0.81	1.09	0.30	[0.55, 1.20]
Traumatic exposure (BTQ)	0.17	0.05	1.18	11.87	<0.01	[1.08, 1.30]
ACE	0.09	0.04	1.09	5.52	0.02	[1.02, 1.18]
S-UPPS-P negative urgency	0.04	0.04	1.04	1.39	0.24	[0.97, 1.12]
S-UPPS-P lack of premeditation	−0.03	0.04	0.97	0.37	0.55	[0.90, 1.06]
S-UPPS-P lack of perseverance	0.03	0.05	1.03	0.33	0.57	[0.94, 1.13]
S-UPPS-P sensation seeking	0.07	0.04	1.08	3.72	0.05	[1.00, 1.16]
S-UPPS-P positive urgency	0.04	0.04	1.05	1.49	0.22	[0.97, 1.12]
Delay discounting ($100)	0.14	0.09	1.15	2.36	0.13	[0.96, 1.37]
Delay discounting ($1,000)	0.03	0.10	1.03	0.08	0.77	[0.85, 1.25]
Combined significant predictors^b^						
Traumatic exposure (BTQ)	0.12	0.06	1.13	4.16	0.04	[1.01, 1.27]
ACE	0.04	0.05	1.04	0.69	0.41	[0.95, 1.14]
S-UPPS-P sensation seeking	0.06	0.04	1.06	1.92	0.17	[0.98, 1.14]

*Note.* OR = odds ratio; CI = confidence interval; BTQ = Brief Trauma Questionnaire; ACE = Adverse Childhood Experience Questionnaire; S-UPPS-P = Shortened Urgency-Premeditation-Perseverance-Sensation Seeking-Positive Urgency Impulsive Behavior Scale.

^a^ Individual predictor models: only one variable was included as a predictor in each of these models. No covariates were included.

^b^ Combined predictor model: only the variables listed were included as predictors in this model.

Lifetime trauma exposure (β = 0.15, *p* = 0.02) and S-UPPS-P lack of premeditation (β = 0.13, *p* = 0.05) emerged as significant individual predictors of number of lifetime overdoses. Combining these two variables into one model explained 2.4% of the variance in total number of lifetime overdoses (*R*^2^ = 0.02, *F* = 3.93, *p* = 0.02). In the combined model, lifetime trauma exposure (β = 0.14, *p* = 0.04) remained a significant predictor of lifetime number of overdoses, while S-UPPS-P lack of premeditation (β = 0.12, *p* = 0.07) was not a significant predictor (Table 3).

**Table 3.** Predictors of Number of Lifetime Overdoses in Overdose Only Sample Using Linear Regression.

	β	*B*	*SE*	*t*	*p*
Individual predictors^a^					
Age	0.09	<0.01	<0.01	−1.46	0.15
Sex	−0.05	−0.04	0.06	−0.76	0.45
Traumatic exposure (BTQ)	0.15	0.03	0.01	2.37	0.02
ACE	0.11	0.02	0.01	1.79	0.07
S-UPPS-P negative urgency	−0.04	−0.01	0.01	−0.61	0.54
S-UPPS-P lack of premeditation	0.13	0.02	0.01	1.98	0.05
S-UPPS-P lack of perseverance	0.02	0.01	0.01	0.38	0.71
S-UPPS-P sensation seeking	0.05	0.01	0.01	0.79	0.43
S-UPPS-P positive urgency	0.08	0.01	0.01	1.29	0.20
Delay discounting ($100)	0.03	0.01	0.03	0.49	0.63
Delay discounting ($1,000)	0.05	0.02	0.03	0.68	0.50
Combined significant predictors^b^					
Traumatic exposure (BTQ)	0.14	0.03	0.01	2.11	0.04
S-UPPS-P lack of premeditation	0.12	0.02	0.01	1.84	0.07

*Note.* BTQ = Brief Trauma Questionnaire; ACE = Adverse Childhood Experience Questionnaire; S-UPPS-P = Shortened Urgency-Premeditation-Perseverance-Sensation Seeking-Positive Urgency Impulsive Behavior Scale.

^a^ Individual predictor models: only one variable was included as a predictor in each of these models. No covariates were included.

^b^ Combined predictor model: only the variables listed were included as predictors in this model.

### Sex Differences

Supplementary Table 6 provides a comprehensive comparison of baseline characteristics by sex. No significant differences were observed in age, ethnicity, financial stability, overdose history, delay discounting, lifetime traumatic exposure, or S-UPPS-P lack of perseverance and positive urgency subscales. Relative to male participants, female participants reported higher levels of education and gender diversity. Male and female participants also differed significantly on ACEs and some measures of impulsivity (S-UPPS-P negative urgency, lack of premeditation, and sensation seeking).

#### Predicting Overdose

We tested the moderating role of sex in the relationship between individual baseline characteristics and overdose outcomes (Supplementary Tables 6a and 6b). Sex was a significant moderator of the relationship between ACEs (*B* = −0.22, *p* < 0.01), lifetime traumatic exposure (*B* = −0.21, *p* = 0.03), and history of overdose. The effect of lifetime traumatic exposure on history of overdose was significant for female participants (*B* = 0.28, *p* < 0.01) and nonsignificant for male participants (*B* = 0.07, *p* = 0.32). Likewise, the effect of ACEs on history of overdose was significant for female participants (*B* = 0.18, *p* < 0.01) and nonsignificant for male participants (*B* = −0.04, *p* = 0.53).

Among individuals with a history of overdose, sex was a significant moderator of the relationship between age (*B* = −0.01, *p* = 0.027), ACEs (*B* = −0.04, *p* = 0.05), and number of lifetime overdoses. The effect of age on number of lifetime overdoses was nonsignificant for female participants (*B* < 0.01, *p* = 0.58) and significant for male participants (*B* = −0.01, *p* = 0.01). The effect of ACEs on number of overdoses was significant for female participants (*B* = 0.04, *p* = 0.01) and nonsignificant for male participants (*B* < −0.01, *p* = 0.63).

### Site Differences

Comparisons were conducted across sites for all demographic, clinical predictor, and outcome variables. Full details are provided in the Supplementary Materials.

#### Demographics Across Sites

There were significant differences in demographic characteristics between the participants from the three distinct sites. On average, participants at Heartwood were older (*M* = 37.5 years, *SD* = 10.2) than Red Fish (*M* = 34.8 years, *SD* = 10.8) and St. Joseph's (*M* = 34.3 years, *SD* = 11.5) participants. There was a significant difference in sex between Heartwood and the other sites; however, this was expected given that Heartwood is a women's-only treatment centre. In terms of level of education, 57.8% of participants at Heartwood reported having completed some university/college compared to 42.7% of St. Joseph's and 30.4% of Red Fish participants. Nearly one in three (27%) Red Fish participants indicated they currently had difficulty paying bills, compared to 38.3% of Heartwood and 39.8% of St. Joseph's participants.

#### Comparing Trauma and Impulsivity Across Sites

There were significant differences in the predictor and outcome variables between the sites. Significant differences were observed on four of the five S-UPPS-P dimensions of impulsivity: negative urgency (*M*_Red Fish_ = 10.8, *M*_Heartwood_ = 12.1, *M*_St. Joseph’s_ = 11.3), lack of premeditation (*M*_Red Fish_ = 12.5, *M*_Heartwood_ = 11.5, *M*_St. Joseph’s_ = 12.0), sensation seeking (*M*_Red Fish_ = 11.5, *M*_Heartwood_ = 10.3, *M*_St. Joseph’s_ = 10.7), and positive urgency (*M*_Red Fish_ = 9.8, *M*_Heartwood_ = 9.4, *M*_St. Joseph’s_ = 10.7). There were no differences in measures assessing delay discounting. Participants from all sites reported similar levels of ACEs and lifetime trauma. Finally, significant differences existed between the sites in the percentage of participants reporting a history of overdose: 74.5% at Red Fish, 61.2% at Heartwood, and 46.6% at St. Joseph's. However, there was no difference in the average number of overdoses reported across the sites.

#### Predicting Overdose Across Sites

##### Red Fish

Lifetime traumatic exposure (*B* = 0.23, *p* < 0.01) and S-UPPS-P positive urgency (*B* = 0.11, *p* = 0.04) were significant individual predictors of endorsing a history of overdose. Combining these variables into one multivariate model predicted 76.1% of group status correctly (Cox and Snell *R*^2^ = 0.06, Nagelkerke *R*^2^ = 0.10). S-UPPS-P lack of premeditation (β = 0.19, *p* = 0.02) was a significant individual predictor of number of lifetime overdoses.

##### Heartwood

ACEs (*B* = 0.19, *p* = 0.01) and lifetime traumatic exposure (*B* = 0.26, *p* = 0.02) were significant individual predictors of endorsing a history of overdose. Combining these variables into one multivariate model predicted 65.1% of group status correctly (Cox and Snell *R*^2^ = 0.08, Nagelkerke *R*^2^ = 0.11). Delay discounting $1,000 (β = 0.13, *p* = 0.04) was a significant individual predictor and explained 7.5% of the variance in number of lifetime overdoses (*R*^2^ = 0.08, *F* = 4.64, *p* = 0.04).

##### St. Joseph's

Sex (*B* = 0.91, *p* = 0.03) was the only significant individual predictor of endorsing a history of overdose. Sex (β = −0.39, *p* = 0.01) and lifetime traumatic exposure (β = 0.37, *p* = 0.02) were significant individual predictors of number of lifetime overdoses. Combining these two variables into one model explained 35.5% of the variance in number of lifetime overdoses (*R*^2^ = 0.36, *F* = 12.54, *p* < 0.01). Both sex (β = −0.51, *p* < 0.01) and lifetime traumatic exposure (β = 0.46, *p* < 0.01) were significant predictors of number of lifetime overdoses.

## Discussion

The ROAR CANADA study rigorously assessed people recruited from three clinical sites that provide services to high-needs patients with SCD, a population that is hard-to-reach and often excluded from research. In this first report, we describe methodology and provide some basic information on the profile of the population studied, focusing on history of overdose, sociodemographic factors, and two relevant transdiagnostic domains, trauma history and impulsivity.

The patient populations at each site were broadly comparable in their demographics, suggesting commonalities across centres and regions in the demographics of those who access treatment. Male and female participants were relatively comparable on most variables, including overdose, with some differences in impulsivity and ACEs. There were some between-site differences, which may reflect regional patterns in substance use and overdose. BC has the highest rate of drug overdose-related hospitalizations and overdose deaths in Canada, over two times greater than any other province.^1,3^

When comparing those with or without any history of overdose, there were no differences in demographics or most dimensions of impulsivity. Individuals in the study reported high levels of adverse childhood events, extensive lifetime trauma histories, and high levels of impulsivity. The history of reported overdoses was extremely high, with overall 64.7% reporting any overdose history. Participants reported 7.6 lifetime overdoses on average. Lifetime history of trauma as assessed by the BTQ was significantly associated with both overdose history and number of overdoses. This finding was consistent across all sites, independent of demographic variables, in line with literature demonstrating a stronger association between lifetime trauma exposure and opioid addiction than childhood trauma.^47^ Of note, the effect of trauma and adversity on overdose was amplified for female participants indicating some sex-based differences in risk factors. This coincides with findings showing a relationship between ACEs and opioid misuse in women but not men,^48^ and broader literature suggesting different pathways to substance misuse between women and men.^49–52^ Impulsivity was associated with overdose in the bivariate analysis, but it was also related to trauma history and was no longer significant in the multivariate analysis. This suggests that the observed significant associations between impulsivity and overdose were related to a history of trauma. The mechanisms by which these factors may be related were not investigated in this paper; however, other research has indicated that emotion dysregulation may be an important avenue for exploration.^53,54^

The study reveals lifetime trauma to be closely related to experiencing overdoses for patients with SCD. These findings highlight the importance of prevention and trauma-informed care and interventions that target trauma-related outcomes.^55,56^ While trauma-informed practices are now presumed to be a standard component of care in mental health and substance use treatment, they are frequently not implemented. Early intervention to address trauma in vulnerable populations may help prevent substance use, mental disorders, and overdoses. It is less clear how trauma history relates to overdose outcomes, such as whether overall SUD acuity is higher or whether individuals with a trauma history are more likely to engage in higher-risk behaviours as part of a negative reinforcement cycle to alleviate distress.

### Limitations and Future Directions

This study has some limitations. First, analyses are based primarily on patient-reported outcomes. Second, the BTQ measures trauma throughout the lifetime and trauma exposure may have been recent, potentially closer to overdoses, when participants were already struggling with SUDs, or it may have distantly preceded SUD onset. Temporality of the trauma relative to substance use onset and overdose may be relevant to assess how trauma may increase risk of overdose. Interestingly, the ACE Questionnaire did not predict overdose history in our multivariate analysis, nor number of overdoses in the bivariate analysis, in contrast with other literature.^57,58^ However, those studies did not specifically target SCD; moreover, they did not assess lifetime exposure to trauma in addition to ACEs and childhood trauma. Our study suggests that lifetime traumatization may have a stronger association with overdose in a SCD population than trauma and adversity in childhood alone. In previous work with a similar population, we found that childhood trauma increased the risk of adult re-traumatization,^29^ but overall, the relationship between ACEs, trauma, and overdose needs to be further examined in people with SCD. Overall, there is a greater need for substance use and mental health research that captures the diversity and high comorbidities in this complex patient population.

## Conclusions

Patients suffering from SCD, specifically those supported in tertiary treatment centres, have an elevated risk of experiencing a substantial number of overdoses. This study assessed common transdiagnostic factors associated with SCD including demographics, impulsivity, and trauma history. Impulsivity was associated with overdose in the bivariate analysis, but was also related to trauma history and was no longer significant in the multivariate analysis. In the SCD population, ACEs are common, and patients display substantial impulsivity overall, but among persistent transdiagnostic factors, lifetime history of trauma (not specifically childhood adversity) emerged as the factor most closely related to overdose. Sex-based differences in risk factors emerged indicating that ACEs and lifetime trauma together may increase overdose risk in women more than men. The prevalence of trauma in such a heterogenous sample and its status as the only significant unique predictor of overdose underscores its impact in the lives of individuals with SCD. As we advance our understanding of the complex interplay between factors related to overdose risk, trauma is a critical element to consider for future studies and in concurrent disorder treatment.

## Supplemental Material

sj-docx-1-cpa-10.1177_07067437251315516 - Supplemental material for Enhancing Integrated Treatment Programs for Severe Concurrent Substance Use and Mental Disorders: Insights on Overdose from the ROAR CANADA Project: Améliorer les programmes de traitement intégré pour les troubles mentaux et les troubles liés à l’usage de substances psychoactives graves et concomitants : aperçu de la problématique des surdoses dans le cadre du projet ROAR CANADA Supplemental material, sj-docx-1-cpa-10.1177_07067437251315516 for Enhancing Integrated Treatment Programs for Severe Concurrent Substance Use and Mental Disorders: Insights on Overdose from the ROAR CANADA Project: Améliorer les programmes de traitement intégré pour les troubles mentaux et les troubles liés à l’usage de substances psychoactives graves et concomitants : aperçu de la problématique des surdoses dans le cadre du projet ROAR CANADA by Christian G. Schütz, Tonia Nicholls, Laura Schmid, Sydney Penner, Myra Massey, Karina A. Thiessen, Stefanie Todesco, Reza Rafizadeh, Kiefer Cowie, Sabrina K. Syan and James MacKillop in The Canadian Journal of Psychiatry

## References

[1] Belzak L Halverson J . Evidence synthesis – the opioid crisis in Canada: a national perspective. Health Promot Chronic Dis Prev Can. 2018;38(6):224–233. doi:10.24095/hpcdp.38.6.02 29911818 PMC6034966

[2] Crabtree A Lostchuck E Chong M Shapiro A Slaunwhite A . Toxicology and prescribed medication histories among people experiencing fatal illicit drug overdose in British Columbia, Canada. CMAJ. 2020;192(34):E967–E972. doi:10.1503/cmaj.200191

[3] Federal, Provincial, and Territorial Special Advisory Committee on the Epidemic of Opioid Overdoses. Opioid- and stimulant-related harms in Canada. Ottawa: Public Health Agency of Canada; 2023. Available from: https://health-infobase.canada.ca/substance-related-harms/opioids-stimulants/.

[4] Palis H Xavier C Dobrer S , et al. Concurrent use of opioids and stimulants and risk of fatal overdose: a cohort study. BMC Public Health. 2022;22(1):2084. doi:10.1186/s12889-022-14506-w 36380298 PMC9664696

[5] Palis H Bélair M Hu K Tu A Buxton J Slaunwhite A . Overdose deaths and the COVID-19 pandemic in British Columbia, Canada. Drug Alcohol Rev. 2022;41(4):912–917. doi:10.1111/dar.13424 34908203

[6] Palis H Gan W Xavier C , et al. Association of opioid and stimulant use disorder diagnoses with fatal and nonfatal overdose among people with a history of incarceration. JAMA Netw Open. 2022;5(11):e2243653. doi:10.1001/jamanetworkopen.2022.43653 PMC9685494 36416821

[7] Ritchie H Arriagada P Roser M . Opioids, cocaine, cannabis, and other illicit drugs. 2022. Available from: https://ourworldindata.org/illicit-drug-use.

[8] Snowdon J . Drug overdose death rates in different countries: who should be alarmed? Australas Psychiatry. 2022;30(1):26–30. doi:10.1177/10398562221075192 35236130

[9] Public Health Agency of Canada. Modelling opioid-related deaths during the overdose crisis. Ottawa: Public Health Agency of Canada; 2023. Available from: https://www.canada.ca/en/health-canada/services/opioids/data-surveillance-research/modelling.html.

[10] Keen C Kinner SA Young JT , et al. Prevalence of co-occurring mental illness and substance use disorder and association with overdose: a linked data cohort study among residents of British Columbia, Canada. Addiction. 2022;117(1):129–140. doi:10.1111/add.15580 34033179

[11] Rush BR Bassani DG Urbanoski KA Castel S . Influence of co-occurring mental and substance use disorders on the prevalence of problem gambling in Canada. Addiction. 2008;103(11):1847–1856. doi:10.1111/j.1360-0443.2008.02338.x 19032535

[12] Keen C Young JT Borschmann R Kinner SA . Non-fatal drug overdose after release from prison: a prospective data linkage study. Drug Alcohol Depend. 2020;206:107707. doi:10.1016/j.drugalcdep.2019.107707 31757517

[13] Bohnert ASB Ilgen MA Ignacio RV McCarthy JF Valenstein M Blow FC . Risk of death from accidental overdose associated with psychiatric and substance use disorders. Am J Psychiatry. 2012;169(1):64–70. doi:10.1176/appi.ajp.2011.10101476 21955932

[14] Wiktorowicz M Abdulle A Di Pierdomenico K Boamah SA . Models of concurrent disorder service: policy, coordination, and access to care. Front Psychiatry. 2019;10:61. doi:10.3389/fpsyt.2019.00061 30837903 PMC6389671

[15] Danilewitz M Bahji A Lamba W Chopra N George TP . Concurrent disorders management in psychiatric care: opportunities and challenges. Canadian Journal of Addiction. 2021;12(3):7–9. doi:10.1097/CXA.0000000000000122

[16] Khan S . Concurrent mental and substance use disorders in Canada. Health Rep. 2017;28(8):3–8. 29044442

[17] Drake RE Mercer-McFadden C Mueser KT McHugo GJ Bond GR . Review of integrated mental health and substance abuse treatment for patients with dual disorders. Schizophr Bull. 1998;24(4):589–608. doi:10.1093/oxfordjournals.schbul.a033351 9853791

[18] Drake RE Luciano AE Mueser KT , et al. Longitudinal course of clients with co-occurring schizophrenia-Spectrum and substance use disorders in urban mental health centers: a 7-year prospective study. Schizophr Bull. 2016;42(1):202–211. doi:10.1093/schbul/sbv110 26294706 PMC4681561

[19] Mueser KT Torrey WC Lynde D Singer P Drake RE . Implementing evidence-based practices for people with severe mental illness. Behav Modif. 2003;27(3):387–411. doi:10.1177/0145445503027003007 12841590

[20] McGovern MP Lambert-Harris C Gotham HJ Claus RE Xie H . Dual diagnosis capability in mental health and addiction treatment services: an assessment of programs across multiple state systems. Adm Policy Mental Health. 2014;41(2):205–214. doi:10.1007/s10488-012-0449-1 PMC3594447 23183873

[21] Park TW Lin LA Hosanagar A Kogowski A Paige K Bohnert ASB . Understanding risk factors for opioid overdose in clinical populations to inform treatment and policy. J Addict Med. 2016;10(6):369–381. doi:10.1097/ADM.0000000000000245 27525471

[22] McKee SA Harris GT Cormier CA . Implementing residential integrated treatment for co-occurring disorders. J Dual Diagn. 2013;9(3):249–259. doi:10.1080/15504263.2013.807073 23976887 PMC3746518

[23] Chow CM Wieman D Cichocki B Qvicklund H Hiersteiner D . Mission impossible: treating serious mental illness and substance use co-occurring disorder with integrated treatment: a meta-analysis. Ment Health Subst Use. 2013;6(2):150–168. doi:10.1080/17523281.2012.693130

[24] Lee-Cheong S Vazirian S Nie G , et al. Development and challenges of Canada’s largest inpatient program for patients with severe concurrent disorders. Can J Addict. 2021;12(4):38–47. doi:10.1097/CXA.0000000000000127

[25] Schütz C Linden IA Torchalla I Li K Al-Desouki M Krausz M . The Burnaby treatment center for mental health and addiction, a novel integrated treatment program for patients with addiction and concurrent disorders: results from a program evaluation. BMC Health Serv Res. 2013;13(1):288. doi:10.1186/1472-6963-13-288 23895592 PMC3733750

[26] Szerman N Parro-Torres C Didia-Attas J El-Guebaly N . Dual disorders: addiction and other mental disorders. Integrating mental health. In: Javed A Fountoulakis KN , editors. Advances in psychiatry. Cham: Springer International Publishing; 2019. p. 109–127. Available from: http://link.springer.com/10.1007/978-3-319-70554-5_7.

[27] Hakobyan S Vazirian S Lee-Cheong S Krausz M Honer WG Schutz CG . Concurrent disorder management guidelines. Systematic review. J Clin Med. 2020;9(8):2406. doi:10.3390/jcm9082406 32731398 PMC7463987

[28] Clous E Beerthuizen K Ponsen KJ Luitse J Olff M Goslings C . Trauma and psychiatric disorders: a systematic review. J Trauma Acute Care Surg. 2017;82(4):794–801. doi:10.1097/TA.0000000000001371 28129262

[29] Edalati H Krank MD Schütz CG . Childhood maltreatment and perceived stress in individuals with concurrent psychiatric disorders. J Aggress Maltreat Trauma. 2020;29(1):22–37. doi:10.1080/10926771.2019.1595802

[30] Moeller FG Barratt ES Dougherty DM Schmitz JM Swann AC . Psychiatric aspects of impulsivity. Am J Psychiatry. 2001;158(11):1783–1793. doi:10.1176/appi.ajp.158.11.1783 11691682

[31] Saddichha S Schuetz C . Impulsivity in remitted depression: a meta-analytical review. Asian J Psychiatr. 2014;9:13–16. doi:10.1016/j.ajp.2014.02.003 24813029

[32] Fusar-Poli P Solmi M Brondino N , et al. Transdiagnostic psychiatry: a systematic review. World Psychiatry. 2019;18(2):192–207. doi:10.1002/wps.20631 31059629 PMC6502428

[33] Spalletta G Janiri D Piras F Sani G . Childhood trauma in mental disorders: a comprehensive approach. Cham: Springer International Publishing; 2020. Available from: https://link.springer.com/10.1007/978-3-030-49414-8.

[34] Mauritz MW Goossens PJJ Draijer N Van Achterberg T . Prevalence of interpersonal trauma exposure and trauma-related disorders in severe mental illness. Eur J Psychotraumatol. 2013;4(1):19985. doi:10.3402/ejpt.v4i0.19985 PMC3621904 23577228

[35] Ramírez-Martín A Ramos-Martín J Mayoral-Cleries F Moreno-Küstner B Guzman-Parra J . Impulsivity, decision-making and risk-taking behaviour in bipolar disorder: a systematic review and meta-analysis. Psychol Med. 2020;50(13):2141–2153. doi:10.1017/S0033291720003086 32878660

[36] Ouzir M . Impulsivity in schizophrenia: a comprehensive update. Aggress Violent Behav. 2013;18(2):247–254. doi:10.1016/j.avb.2012.11.014

[37] Van Draanen J Tsang C Mitra S , et al. Mental disorder and opioid overdose: a systematic review. Soc Psychiatry Psychiatr Epidemiol. 2022;57(4):647–671. doi:10.1007/s00127-021-02199-2 34796369 PMC8601097

[38] Van Amsterdam J Van Den Brink W . Explaining the high mortality among opioid-cocaine co-users compared to opioid-only users. A systematic review. J Addict Dis. 2024:1–11. doi:10.1080/10550887.2024.2331522 38504419

[39] Byrne CJ Sani F Thain D Fletcher EH Malaguti A . Psychosocial factors associated with overdose subsequent to illicit drug use: a systematic review and narrative synthesis. Harm Reduct J. 2024;21(1):81. doi:10.1186/s12954-024-00999-8 38622647 PMC11017611

[40] Lake S Hayashi K Milloy M-J , et al. Associations between childhood trauma and non-fatal overdose among people who inject drugs. Addict Behav. 2015;43:83–88. doi:10.1016/j.addbeh.2014.12.014 25588793 PMC4304975

[41] Keen C Kinner SA Young JT , et al. Periods of altered risk for non-fatal drug overdose: a self-controlled case series. Lancet Public Health. 2021;6(4):e249–e259. doi:10.1016/S2468-2667(21)00007-4 33773635

[42] Lyden JR Xu S Narwaney KJ Glanz JM Binswanger IA . Opioid overdose risk following hospital discharge among individuals prescribed long-term opioid therapy: a risk interval analysis. J Gen Intern Med. 2023;38(11):2560–2567. doi:10.1007/s11606-022-08014-1 36697930 PMC9876414

[43] Felitti VJ Anda RF Nordenberg D , et al. Relationship of childhood abuse and household dysfunction to many of the leading causes of death in adults. Am J Prev Med. 1998;14(4):245–258. doi:10.1016/S0749-3797(98)00017-8 9635069

[44] Schnurr P Vielhauer M Weathers F Findler M . Brief Trauma Questionnaire. 2012 [accessed 2024 Jan 23]. https://doi.apa.org/getdoi.cfm?doi=10.1037/t07488-000.

[45] Cyders MA Littlefield AK Coffey S Karyadi KA . Examination of a short English version of the UPPS-P impulsive behavior scale. Addict Behav. 2014;39(9):1372–1376. doi:10.1016/j.addbeh.2014.02.013 24636739 PMC4055534

[46] Kirby KN Petry NM Bickel WK . Monetary Choice Questionnaire. 2012 [accessed 2024 Jan 23]. https://doi.apa.org/getdoi.cfm?doi=10.1037/t10044-000.

[47] Garami J Valikhani A Parkes D , et al. Examining perceived stress, childhood trauma and interpersonal trauma in individuals with drug addiction. Psychol Rep. 2019;122(2):433–450. doi:10.1177/0033294118764918 29569991

[48] Williams JR Girdler S Williams W Cromeens MG . The effects of co-occurring interpersonal trauma and gender on opioid use and misuse. J Interpers Violence. 2021;36(23–24):NP13185–NP13205. doi:10.1177/0886260519900309 32054388

[49] Boyd J Collins AB Mayer S Maher L Kerr T McNeil R . Gendered violence and overdose prevention sites: a rapid ethnographic study during an overdose epidemic in Vancouver, Canada. Addiction. 2018;113(12):2261–2270. doi:10.1111/add.14417 30211453 PMC6400212

[50] Thumath M Humphreys D Barlow J , et al. Overdose among mothers: the association between child removal and unintentional drug overdose in a longitudinal cohort of marginalised women in Canada. Int J Drug Policy. 2021;91:102977. doi:10.1016/j.drugpo.2020.102977 33129662 PMC8081759

[51] Danielson CK Amstadter AB Dangelmaier RE Resnick HS Saunders BE Kilpatrick DG . Trauma-related risk factors for substance abuse among male versus female young adults. Addict Behav. 2009;34(4):395–399. doi:10.1016/j.addbeh.2008.11.009 19110381 PMC2704020

[52] Bagley SM Gai MJ Earlywine JJ Schoenberger SF Hadland SE Barocas JA . Incidence and characteristics of nonfatal opioid overdose among youths aged 11 to 24 years by sex. JAMA Netw Open. 2020;3(12):e2030201. doi:10.1001/jamanetworkopen.2020.30201 PMC7747019 33331919

[53] Weiss NH Kiefer R Goncharenko S , et al. Emotion regulation and substance use: a meta-analysis. Drug Alcohol Depend. 2022;230:109131. doi:10.1016/j.drugalcdep.2021.109131 34864568 PMC8714680

[54] Weiss NH Tull MT Viana AG Anestis MD Gratz KL . Impulsive behaviors as an emotion regulation strategy: examining associations between PTSD, emotion dysregulation, and impulsive behaviors among substance dependent inpatients. J Anxiety Disord. 2012;26(3):453–458. doi:10.1016/j.janxdis.2012.01.007 22366447 PMC3305816

[55] Shier ML Turpin A . Trauma-informed organizational dynamics and client outcomes in concurrent disorder treatment. Res Soc Work Pract. 2022;32(1):92–105. doi:10.1177/10497315211013908

[56] Torchalla I Nosen L Rostam H Allen P . Integrated treatment programs for individuals with concurrent substance use disorders and trauma experiences: a systematic review and meta-analysis. J Subst Abuse Treat. 2012;42(1):65–77. doi:10.1016/j.jsat.2011.09.001 22035700

[57] Asheh AM Courchesne-Krak N Kepner W Marienfeld C . Adverse childhood experiences are associated with history of overdose among patients presenting for outpatient addiction care. J Addict Med. 2023;17(3):333–338. doi:10.1097/ADM.0000000000001126 37267182 PMC10241414

[58] Tschampl CA Canuto M De Jesús D , et al. Adverse childhood experiences are associated with increased overdose risk in predominately Latinx adults seeking treatment for substance use disorders. Front Psychiatry. 2022;13:987085. doi:10.3389/fpsyt.2022.987085 36590627 PMC9798211

