# Supplementary material for: Enhancing Integrated Treatment Programs for Severe Concurrent Substance Use and Mental Disorders: Insights on Overdose from the ROAR CANADA Project: Améliorer les programmes de traitement intégré pour les troubles mentaux et les troubles liés à l’usage de substances psychoactives graves et concomitants : aperçu de la problématique des surdoses dans le cadre du projet ROAR CANADA
Source: Can J Psychiatry. 2025 Feb 3;70(8):600–10. doi: 10.1177/07067437251315516 (PMC11795578; doi:10.1177/07067437251315516)
Supplement: sj-docx-1-cpa-10.1177_07067437251315516 - Supplemental material for Enhancing Integrated Treatment Programs for Severe Concurrent Substance Use and Mental Disorders: Insights on Overdose from the ROAR CANADA Project: Améliorer les programmes de traitement intégré pour les troubles mentaux et les tro [file sj-docx-1-cpa-10.1177_07067437251315516.docx]

**Supplementary Material**

**Supplemental Table 1.**

*Percentage of Participants Endorsing Traumatic Exposure*

| Traumatic Exposure | All Participants (*N* = 450) | No Overdose History  (*n* = 159) | History of Overdose  (*n* = 291) | *t* | *p* | Phi |
| --- | --- | --- | --- | --- | --- | --- |
| War | 4.2% | 2.5% | 5.2% | 1.71 | .19 | 0.06 |
| Serious Accident | 39.6% | 29.6% | 45.0% | 10.12 | < .01 | 0.15 |
| Natural or Technological Disaster | 16.7% | 18.2% | 15.8% | 0.53 | .47 | -0.04 |
| Life Threatening Illness | 18.2% | 13.2% | 21.0% | 4.09 | .04 | 0.10 |
| Childhood Physical Assault | 43.6% | 34.0% | 48.8% | 9.05 | < .01 | 0.15 |
| Adult Physical Assault | 59.6% | 50.9% | 64.3% | 7.33 | < .01 | 0.13 |
| Unwanted Sexual Contact | 57.8% | 51.6% | 61.2% | 3.30 | .07 | 0.09 |
| Serious Injury or Threat of Death | 51.3% | 45.3% | 54.6% | 2.71 | .10 | 0.08 |
| Violent Loss of Loved One | 32.4% | 27.7% | 35.1% | 2.33 | .13 | 0.07 |
| Witnessed serious injury or death of another person | 42.4% | 40.3% | 43.6% | 0.13 | .72 | 0.02 |

**Supplemental Table 2.**

*Participant Characteristics by Site*

|  | All Participants  (*N* = 450) | Red Fish  (*n* = 231; 51.1%) | Heartwood (*n* = 116; 25.7%) | St. Joseph’s  (*n* = 103; 22.8%) | *p* |
| --- | --- | --- | --- | --- | --- |
| **Demographics** |  |  |  |  |  |
| Age | 35.4 (10.9) | 34.8 (10.8) | 37.5 (10.2) | 34.3 (11.5) | .05 |
| Biological Sex (% female) | 54.6% | 41.1% | 100.0% | 35% | < .01 |
| Gender (% cisgender) | 92.3% | 93.5% | 91.4% | 92.2% | .66 |
| Ethnicity (% European / white) | 58.2% | 55.4% | 64.7% | 58.3% | .26 |
| Education (% university/college) | 40.2% | 30.4% | 57.8.% | 42.7% | < .01 |
| Financial Status (% difficulty paying bills) | 32.8% | 27.0% | 38.3% | 39.8% | .03 |
| **Overdose** |  |  |  |  |  |
| History of Overdose (% yes) | 64.7% | 74.5% | 61.2% | 46.6% | < .01 |
| Number of Overdoses | 7.6 (12.9) | 8.5 (14.5) | 6.0 (10.1) | 7.1 (10.2) | .38 |
| **Trauma and Adversity** |  |  |  |  |  |
| Traumatic Exposure (BTQ) | 3.8 (2.2) | 3.6 (2.2) | 4.2 (1.9) | 3.9 (2.6) | .08 |
| Adverse Childhood Experiences (ACE) | 4.6 (2.8) | 4.4 (2.8) | 5.1 (2.7) | 4.6 (2.7) | .07 |
| **Impulsivity** |  |  |  |  |  |
| Negative Urgency | 11.2 (3.0) | 10.8 (3.1) | 12.1 (2.6) | 11.3 (3.0) | < .01 |
| Lack of Perseverance | 12.4 (2.3) | 12.5 (2.2) | 12.0 (2.5) | 12.5 (2.1) | .11 |
| Lack of Premeditation | 12.1 (2.5) | 12.5 (2.4) | 11.5 (2.6) | 12.0 (2.5) | < .01 |
| Sensation Seeking | 11.0 (2.9) | 11.5 (2.8) | 10.3 (2.5) | 10.7 (3.2) | < .01 |
| Positive Urgency | 9.9 (3.0) | 9.8 (3.1) | 9.4 (2.8) | 10.7 (3.0) | .01 |
| Delay Discounting ($100) | -1.5 (1.2) | -1.5 (1.3) | -1.5 (1.2) | -1.4 (1.2) | .85 |
| Delay Discounting ($1000) | -1.8 (1.1) | -1.8 (1.1) | -2.0 (1.1) | -1.7 (1.1) | .37 |

*Notes.* BTQ: Brief Trauma Questionnaire; ACE: Adverse Childhood Experience Questionnaire; S-UPPS-P: Shortened Urgency-Premeditation-Perseverance-Sensation Seeking-Positive Urgency Impulsive Behavior Scale; Delay Discounting Task

**Supplemental Table 3a.**

*Predictors of experiencing an overdose in Red Fish sample using a binary logistic regression*

|  | *B* | SE | *OR* | Wald *X*^2^ | *p* | 95% CI |
| --- | --- | --- | --- | --- | --- | --- |
| Individual Predictors^a^ |  |  |  |  |  |  |
| Age | 0.02 | 0.02 | 1.02 | 1.18 | 0.28 | [0.99, 1.05] |
| Sex | -0.12 | 0.31 | 0.89 | 0.15 | 0.70 | [0.49, 1.62] |
| Traumatic Exposure (BTQ) | 0.23 | 0.08 | 1.25 | 8.20 | < 0.01 | [1.07, 1.46] |
| Adverse Childhood Experiences (ACE) | 0.06 | 0.06 | 1.06 | 1.00 | 0.32 | [0.95, 1.19] |
| S-UPPS-P Negative Urgency | 0.06 | 0.06 | 1.06 | 1.08 | 0.30 | [0.95, 1.18] |
| S-UPPS-P Lack of Premeditation | -0.03 | 0.07 | 0.98 | 0.13 | 0.72 | [0.85, 1.12] |
| S-UPPS-P Lack of Perseverance | -0.02 | 0.08 | 0.98 | 0.05 | 0.82 | [0.84, 1.14] |
| S-UPPS-P Sensation Seeking | 0.11 | 0.06 | 1.11 | 3.11 | 0.08 | [0.99, 1.25] |
| S-UPPS-P Positive Urgency | 0.11 | 0.06 | 1.12 | 4.12 | 0.04 | [1.00, 1.25] |
| Delay Discounting ($100) | 0.21 | 0.14 | 1.24 | 2.37 | 0.12 | [0.94, 1.63] |
| Delay Discounting ($1000) | 0.03 | 0.16 | 1.03 | 0.03 | 0.87 | [0.75, 1.40] |
| Combined Significant Predictors^b^ |  |  |  |  |  |  |
| Traumatic Exposure (BTQ) | 0.23 | 0.09 | 1.26 | 7.51 | < 0.01 | [1.07, 1.49] |
| S-UPPS-P Positive Urgency | 0.12 | 0.06 | 1.13 | 4.22 | 0.04 | [1.01, 1.27] |

^a^ Individual predictor models: only one variable was included as a predictor in each of these models. No covariates were included.

^b^ Combined predictor model: only the variables listed were included.

**Supplemental Table 3b.**

*Predictors of number of lifetime overdoses in Red Fish overdose only sample using linear regression*

|  | β | *B* | SE | *t* | *p* |
| --- | --- | --- | --- | --- | --- |
| Individual Predictors^a^ |  |  |  |  |  |
| Age | -0.13 | -0.01 | <.01 | -1.73 | 0.09 |
| Sex | -0.03 | -0.03 | 0.07 | -0.40 | 0.69 |
| Traumatic Exposure (BTQ) | 0.15 | 0.03 | 0.02 | 1.88 | 0.06 |
| Adverse Childhood Experiences (ACE) | 0.12 | 0.02 | 0.01 | 1.50 | 0.14 |
| S-UPPS-P Negative Urgency | -0.04 | -0.01 | 0.01 | -0.51 | 0.61 |
| S-UPPS-P Lack of Premeditation | 0.19 | 0.04 | 0.02 | 2.31 | 0.02 |
| S-UPPS-P Lack of Perseverance | 0.05 | 0.01 | 0.02 | 0.63 | 0.53 |
| S-UPPS-P Sensation Seeking | -0.05 | -0.01 | 0.02 | -0.66 | 0.51 |
| S-UPPS-P Positive Urgency | 0.05 | 0.01 | 0.01 | 0.61 | 0.55 |
| Delay Discounting ($100) | <.01 | <.01 | 0.03 | -0.03 | 0.98 |
| Delay Discounting ($1000) | -0.02 | -0.01 | 0.04 | -0.19 | 0.85 |

^a^ Individual predictor models: only one variable was included as a predictor in each of these models. No covariates were included.

No combined model was used as there was only one significant individual predictor.

**Supplemental Table 4a.**

*Predictors of experiencing an overdose in Heartwood sample using a binary logistic regression*

|  | *B* | SE | *OR* | Wald *X*^2^ | *p* | 95% CI |
| --- | --- | --- | --- | --- | --- | --- |
| Individual Predictors^a^ |  |  |  |  |  |  |
| Age | 0.01 | 0.02 | 1.01 | 0.07 | 0.79 | [0.97, 1.04] |
| Traumatic Exposure (BTQ) | 0.26 | 0.11 | 1.30 | 5.36 | 0.02 | [1.04, 1.63] |
| Adverse Childhood Experiences (ACE) | 0.19 | 0.08 | 1.21 | 6.38 | 0.01 | [1.04, 1.40] |
| S-UPPS-P Negative Urgency | 0.05 | 0.08 | 1.05 | 0.35 | 0.56 | [0.89, 1.24] |
| S-UPPS-P Lack of Premeditation | -0.08 | 0.09 | 0.92 | 0.87 | 0.35 | [0.78, 1.09] |
| S-UPPS-P Lack of Perseverance | 0.03 | 0.09 | 1.03 | 0.14 | 0.71 | [0,87, 1.22] |
| S-UPPS-P Sensation Seeking | <.01 | 0.09 | 1.00 | <.01 | 0.98 | [0.85, 1.19] |
| S-UPPS-P Positive Urgency | -0.01 | 0.08 | 1.00 | <.01 | 0.95 | [0.86, 1.16] |
| Delay Discounting ($100) | 0.21 | 0.19 | 1.24 | 1.30 | 0.26 | [0.86, 1.79] |
| Delay Discounting ($1000) | 0.12 | 0.20 | 1.12 | 0.33 | 0.57 | [0.76, 1.66] |
| Combined Significant Predictors^b^ |  |  |  |  |  |  |
| Traumatic Exposure (BTQ) | 0.19 | 0.12 | 1.20 | 2.40 | 0.12 | [0.95, 1.52] |
| Adverse Childhood Experiences (ACE) | 0.15 | 0.08 | 1.16 | 3.61 | 0.06 | [1.00, 1.36] |

^a^ Individual predictor models: only one variable was included as a predictor in each of these models. No covariates were included.

^b^ Combined predictor model: only the variables listed were included.

**Supplemental Table 4b.**

*Predictors of number of lifetime overdoses in Heartwood overdose only sample using linear regression*

|  | β | *B* | SE | *t* | *p* |
| --- | --- | --- | --- | --- | --- |
| Individual Predictors^a^ |  |  |  |  |  |
| Age | 0.08 | <.01 | 0.01 | 0.68 | 0.50 |
| Traumatic Exposure (BTQ) | 0.01 | <.01 | 0.03 | 0.11 | 0.91 |
| Adverse Childhood Experiences (ACE) | 0.10 | 0.02 | 0.02 | 0.75 | 0.46 |
| S-UPPS-P Negative Urgency | 0.15 | 0.03 | 0.02 | 1.08 | 0.29 |
| S-UPPS-P Lack of Premeditation | -0.01 | <.01 | 0.02 | -0.09 | 0.93 |
| S-UPPS-P Lack of Perseverance | 0.09 | 0.01 | 0.02 | 0.61 | 0.55 |
| S-UPPS-P Sensation Seeking | 0.14 | 0.03 | 0.03 | 0.96 | 0.34 |
| S-UPPS-P Positive Urgency | 0.22 | 0.04 | 0.02 | 1.60 | 0.12 |
| Delay Discounting ($100) | 0.16 | 0.06 | 0.06 | 1.14 | 0.26 |
| Delay Discounting ($1000) | 0.31 | 0.13 | 0.06 | 2.15 | 0.04 |

^a^ Individual predictor models: only one variable was included as a predictor in each of these models. No covariates were included.

No combined model was used as there was only one significant individual predictors.

**Supplemental Table 5a.**

*Predictors of experiencing an overdose in St. Joseph’s sample using a binary logistic regression*

|  | *B* | SE | *OR* | Wald *X*^2^ | *p* | 95% CI |
| --- | --- | --- | --- | --- | --- | --- |
| Individual Predictors^a^ |  |  |  |  |  |  |
| Age | 0.04 | 0.02 | 1.04 | 3.66 | 0.06 | [1.00, 1.07] |
| Sex | 0.91 | 0.42 | 2.48 | 4.58 | 0.03 | [1.08, 5.69] |
| Traumatic Exposure (BTQ) | 0.14 | 0.08 | 1.15 | 2.80 | 0.09 | [0.98, 1.35] |
| Adverse Childhood Experiences (ACE) | 0.10 | 0.08 | 1.11 | 1.72 | 0.19 | [0.95, 1.30] |
| S-UPPS-P Negative Urgency | 0.11 | 0.07 | 1.12 | 2.43 | 0.12 | [0.97, 1.29] |
| S-UPPS-P Lack of Premeditation | -0.08 | 0.08 | 0.92 | 0.93 | 0.34 | [0.78, 1.09] |
| S-UPPS-P Lack of Perseverance | 0.07 | 0.10 | 1.07 | 0.51 | 0.48 | [0.88, 1.30] |
| S-UPPS-P Sensation Seeking | 0.01 | 0.07 | 1.01 | 0.04 | 0.85 | [0.89, 1.15] |
| S-UPPS-P Positive Urgency | 0.06 | 0.07 | 1.06 | 0.62 | 0.43 | [0.92, 1.22] |
| Delay Discounting ($100) | -0.01 | 0.18 | 0.99 | <.01 | 0.95 | [0.69, 1.42] |
| Delay Discounting ($1000) | -0.02 | 0.19 | 0.98 | 0.01 | 0.93 | [0.67, 1.44] |

^a^ Individual predictor models: only one variable was included as a predictor in each of these models. No covariates were included.

No combined model was used as there was only one significant individual predictor.

**Supplemental Table 5b.**

*Predictors of number of lifetime overdoses in St. Joseph’s overdose only sample using linear regression*

|  | β | *B* | SE | *t* | *p* |
| --- | --- | --- | --- | --- | --- |
| Individual Predictors^a^ |  |  |  |  |  |
| Age | -0.07 | <.01 | 0.01 | -0.47 | 0.64 |
| Sex | -0.39 | -0.39 | 0.13 | -2.90 | 0.01 |
| Traumatic Exposure (BTQ) | 0.37 | 0.07 | 0.03 | 2.52 | 0.02 |
| Adverse Childhood Experiences (ACE) | 0.22 | 0.04 | 0.03 | 1.46 | 0.15 |
| S-UPPS-P Negative Urgency | -0.12 | -0.02 | 0.03 | -0.81 | 0.43 |
| S-UPPS-P Lack of Premeditation | 0.02 | 0.01 | 0.03 | 0.16 | 0.88 |
| S-UPPS-P Lack of Perseverance | -0.19 | -0.05 | 0.04 | -1.25 | 0.22 |
| S-UPPS-P Sensation Seeking | 0.19 | 0.03 | 0.02 | 1.27 | 0.21 |
| S-UPPS-P Positive Urgency | 0.06 | 0.01 | 0.03 | 0.39 | 0.70 |
| Delay Discounting ($100) | 0.01 | 0.01 | 0.07 | 0.08 | 0.94 |
| Delay Discounting ($1000) | -0.02 | -0.01 | 0.07 | -0.14 | 0.89 |
| Combined Significant Predictors^b^ |  |  |  |  |  |
| Sex | -0.51 | -0.49 | 0.12 | -4.05 | < 0.01 |
| Traumatic Exposure (BTQ) | 0.46 | 0.09 | 0.02 | 3.66 | < 0.01 |

^a^ Individual predictor models: only one variable was included as a predictor in each of these models. No covariates were included.

^b^ Combined predictor model: only the variables listed were included.

**Supplemental Table 6.**

*Comparison of baseline characteristics between male and female participants*

|  | All Participants  (*N* = 450) | Male  (*n* = 203) | Female  (*n* = 247) | *t* | *p* | Cohen’s *d* / Phi |
| --- | --- | --- | --- | --- | --- | --- |
| **Demographics** |  |  |  |  |  |  |
| Age | 35.4 (10.9) | 35.4 (11.4) | 35.3 (10.4) | 0.03 | .97 | <.01 |
| Biological Sex (% female) | 54.9% |  |  |  |  |  |
| Gender (% cisgender) | 92.3% | 95.5% | 90.7% | 3.96 | .05 | 0.09 |
| Ethnicity (% European / white) | 58.4% | 61.1% | 56.3% | 1.06 | .30 | 0.05 |
| Education (% university/college) | 40.2% | 31.0% | 47.8% | 12.98 | <.01 | 0.17 |
| Financial Status (% difficulty paying bills) | 32.8% | 30.2% | 35.0% | 1.14 | .29 | -0.05 |
| **Overdose History** |  |  |  |  |  |  |
| History of Overdose (% yes) | 64.7% | 62.1% | 66.8% | 1.09 | .30 | -0.05 |
| Number of Overdoses | 7.6 (12.9) | 7.8 (15.7) | 7.5 (10.3) | -0.21 | .83 | -0.03 |
| **Trauma and Adversity** |  |  |  |  |  |  |
| Traumatic Exposure (BTQ) | 3.8 (2.2) | 3.7 (2.4) | 4.0 (2.1) | 1.04 | 0.30 | 0.10 |
| Adverse Childhood Experiences (ACE) | 4.6 (2.8) | 4.1 (2.6) | 5.0 (2.8) | 3.44 | <.01 | 0.34 |
| **Impulsivity** |  |  |  |  |  |  |
| Negative Urgency (S-UPPS-P) | 11.2 (3.0) | 10.7 (3.0) | 11.7 (2.8) | 3.46 | <.01 | 0.36 |
| Lack of Perseverance (S-UPPS-P) | 12.4 (2.3) | 12.6 (2.1) | 12.2 (2.3) | -1.77 | .08 | -0.18 |
| Lack of Premeditation (S-UPPS-P) | 12.1 (2.5) | 12.5 (2.4) | 11.8 (2.5) | -2.90 | <.01 | -0.30 |
| Sensation Seeking (S-UPPS-P) | 11.0 (2.9) | 11.7 (2.8) | 10.5 (2.8) | -4.14 | <.01 | -0.43 |
| Positive Urgency (S-UPPS-P) | 9.9 (3.0) | 10.0 (3.0) | 9.8 (3.1) | -0.61 | .54 | -0.06 |
| Delay Discounting ($100) | -1.5 (1.2) | -1.4 (1.3) | -1.6 (1.2) | -1.79 | .07 | -0.19 |
| Delay Discounting ($1000) | -1.8 (1.1) | -1.7 (1.2) | -1.9 (1.1) | -1.70 | .09 | -0.18 |

**Supplemental Table 6a.**

*Sex as a moderator of history of overdose using logistic regression*

|  | Predictor (main effect) | | | | Moderator (interaction) | | |
| --- | --- | --- | --- | --- | --- | --- | --- |
|  | *B* | 95% CI | *p* | *B* | | 95% CI | *p* |
| Sex^a^ |  |  |  |  | |  |  |
| Age | 0.02 | [0.44, 0.97] | .18 | < .01 | | [-0.04, 0.04] | .93 |
| Traumatic Exposure (BTQ) | 0.28 | [0.13, 0.42] | < .01 | -0.21 | | [-0.40, -0.18] | .03 |
| Adverse Childhood Experiences (ACE) | 0.18 | [0.08, 0.28] | < .01 | -0.22 | | [-0.37, -0.06] | .01 |
| S-UPPS-P Negative Urgency | 0.01 | [-0.10, 0.11] | .88 | 0.05 | | [-0.09, 0.20] | .46 |
| S-UPPS-P Lack of Premeditation | -0.06 | [-0.18, 0.06] | .33 | 0.09 | | [-0.09, 0.26] | .32 |
| S-UPPS-P Lack of Perseverance | 0.04 | [-0.09, 0.16] | .58 | -0.01 | | [-0.19, 0.18] | .96 |
| S-UPPS-P Sensation Seeking | 0.04 | [-0.07, 0.14] | .47 | 0.11 | | [-0.49, 0.26] | .18 |
| S-UPPS-P Positive Urgency | < .01 | [-0.09, 0.10] | .96 | 0.09 | | [-0.05, 0.24] | .20 |
| Delay Discounting ($100) | 0.18 | [-0.08, 0.44] | .17 | -0.06 | | [-0.42, 0.30] | .74 |
| Delay Discounting ($1000) | 0.02 | [-0.26, 0.31] | .89 | 0.03 | | [-0.36, 0.43] | .87 |

^a^ Covariate

**Supplemental Table 6b.**

*Sex as a moderator of number of overdoses using linear regression*

|  | Predictor (main effect) | | | | Moderator (interaction) | | |  |
| --- | --- | --- | --- | --- | --- | --- | --- | --- |
|  | *B* | 95% CI | *p* | *B* | | 95% CI | *p* | |
| Sex^a^ |  |  |  |  | |  |  | |
| Age | < .01 | [-0.01, 0.01] | 0.58 | -0.01 | | [-0.02, < -.01] | 0.03 | |
| Traumatic Exposure (BTQ) | 0.05 | [0.015, 0.01] | 0.01 | -0.04 | | [-0.10, 0.01] | 0.10 | |
| Adverse Childhood Experiences (ACE) | 0.04 | [0.01, 0.06] | 0.01 | -0.04 | | [-0.09, < -.01] | 0.05 | |
| S-UPPS-P Negative Urgency | < .01 | [-0.03, 0.03] | 0.86 | -0.02 | | [-0.06, 0.02] | 0.29 | |
| S-UPPS-P Lack of Premeditation | 0.02 | [-0.01, 0.05] | 0.15 | 0.01 | | [-0.04, 0.06] | 0.66 | |
| S-UPPS-P Lack of Perseverance | 0.02 | [-0.01, 0.06] | 0.21 | -0.04 | | [-0.09, 0.02] | 0.19 | |
| S-UPPS-P Sensation Seeking | 0.01 | [-0.02, 0.04] | 0.60 | 0.01 | | [-0.03, 0.06] | 0.56 | |
| S-UPPS-P Positive Urgency | 0.02 | [-0.01, 0.05] | 0.12 | -0.02 | | [-0.06, 0.03] | 0.46 | |
| Delay Discounting ($100) | 0.01 | [-0.06, 0.08] | 0.81 | 0.01 | | [-0.09, 0.12] | 0.78 | |
| Delay Discounting ($1000) | 0.07 | [-0.01, 0.15] | 0.08 | -0.10 | | [-0.21, 0.02] | 0.10 | |

^a^ Covariate
